# HAAD: A Quick Algorithm for Accurate Prediction of Hydrogen Atoms in Protein Structures

**DOI:** 10.1371/journal.pone.0006701

**Published:** 2009-08-20

**Authors:** Yunqi Li, Ambrish Roy, Yang Zhang

**Affiliations:** Center for Bioinformatics and Department of Molecular Bioscience, University of Kansas, Lawrence, Kansas, United States of America; Griffith University, Australia

## Abstract

Hydrogen constitutes nearly half of all atoms in proteins and their positions are essential for analyzing hydrogen-bonding interactions and refining atomic-level structures. However, most protein structures determined by experiments or computer prediction lack hydrogen coordinates. We present a new algorithm, HAAD, to predict the positions of hydrogen atoms based on the positions of heavy atoms. The algorithm is built on the basic rules of orbital hybridization followed by the optimization of steric repulsion and electrostatic interactions. We tested the algorithm using three independent data sets: ultra-high-resolution X-ray structures, structures determined by neutron diffraction, and NOE proton-proton distances. Compared with the widely used programs CHARMM and REDUCE, HAAD has a significantly higher accuracy, with the average RMSD of the predicted hydrogen atoms to the X-ray and neutron diffraction structures decreased by 26% and 11%, respectively. Furthermore, hydrogen atoms placed by HAAD have more matches with the NOE restraints and fewer clashes with heavy atoms. The average CPU cost by HAAD is 18 and 8 times lower than that of CHARMM and REDUCE, respectively. The significant advantage of HAAD in both the accuracy and the speed of the hydrogen additions should make HAAD a useful tool for the detailed study of protein structure and function. Both an executable and the source code of HAAD are freely available at http://zhang.bioinformatics.ku.edu/HAAD.

## Introduction

Hydrogen constitutes nearly half of all atoms in protein molecules and plays an important role in controlling the folding kinetics and in stabilizing the native state through hydrophobic interactions and hydrogen bonding [1], [2], [3], [4]. The non-polar hydrogen atoms in alkyl and aromatic groups contribute to hydrophobic interactions, while the polar hydrogen atoms participate directly in hydrogen bonds. Hydrogen atoms mediate a number of important interactions and considering the energetic contribution associated with them is important in studies such as the analysis of ligand-protein and protein-protein interactions [5], [6], ligand screening [7], and structure-based drug design [8], [9]. Moreover, the exact location of hydrogen atoms plays a critical role in developing atomic-level potentials for refining high-resolution protein structures [10], [11], [12], [13], [14] and is essential for interpreting structural features such as bifurcated hydrogen bonds [15]. However, most protein structures solved by X-ray crystallography in the Protein Data Bank (PDB) and structural models generated by computer programs (e.g. SCWRL [16] and MODELLER [17]) lack hydrogen atoms, which necessitates the development of programs that can predict hydrogen positions accurately and quickly.

There are several algorithms dedicated to predicting the positions of hydrogen atoms [18], [19], [20], [21], [22], [23], [24]. In general, hydrogen atoms are first placed using local geometric restraints and then their positions are optimized by conformational search guided by an energy function[18], [20], [21], [22], [23], or by heuristic approaches[19], [24]. For example, WHAT IF [24] determines the position of non-polar hydrogen atoms using fixed bond lengths and bond angles, while for the polar hydrogen atoms, it considers possible hydrogen bonds and the protonation state of each amino acid. REDUCE [19] searches for the most favorable position of hydrogen atoms by a “contact dot” method and samples the atomic “repulsion surface”. MCCE [18] places the non-hydroxyl hydrogen atoms using standard geometric values for the bond lengths and bond angles, while the hydroxyl hydrogen atom positions are optimized by Monte Carlo simulations guided by an energy function consisting of torsion, excluded volume, solvation, and electrostatic terms. HBUILD[20] uses a unique dihedral angle parameter, defined in the CHARMM22 force field, for the placement of hydrogen atoms. Forest and Honig[18] recently compared the accuracy of several hydrogen addition methods, including REDUCE[19], CHARMM (using the HBUILD subroutine)[20], [21], CNS[22], MCCE[18], GROMACS[23] and WHAT IF[24]. Based on a test using seven protein structures solved by X-ray crystallography and neutron diffraction, the authors concluded that REDUCE, WHAT IF and MCCE are among the best methods for placing hydrogen atoms. HBUILD, implemented in the CHARMM package [20], [21], was also shown to have a comparable performance after energy optimization. Despite the good performance of these programs, an algorithm that is of higher prediction accuracy is always desirable for atomic-level structure modeling and drug screening [9]. Especially, for atomic protein structure simulations[25] and atomic force field based protein structure refinement [26], where detailed hydrogen-bonding energy terms have to be calculated at each step of the modeling movements, high-speed determination of hydrogen atom positions is of key importance.

In this work, we develop a new method, called HAAD (Hydrogen Atom ADdition), for quickly constructing hydrogen atoms by combining local geometry restraints and conformational search. The purpose is to reduce steric repulsion and enhance hydrogen bonding networks in the protein structure. On a comprehensive benchmark, we test our method based on three sets of experimental data: high-resolution X-ray crystallography, structures from neutron diffraction, and NOE proton-proton distance restraints. The widely used methods HUBILD and REDUCE are used as a reference for accuracy measurement. The successes or failures of the algorithms in positioning different types of hydrogen atoms are discussed.

### Methodology

There are three kinds of hybrid orbital, i.e. sp3, sp2 and sp, associated with the heavy atoms (C, N, O and S) in proteins [27]. Given the 3D coordinates of the heavy atoms, the spatial orientations of the hybrid orbital can be used to determine the positions of hydrogen atoms (H-atoms). The position of an H-atom connected to a heavy atom is determined relative to other heavy atoms connected to the same central heavy atom. Basically, if the heavy atom has an sp3 hybrid orbital, the four connected atoms tend to form a tetrahedron centered at this heavy atom; if it has a sp2 hybrid orbital, the three atoms connected to it tend to form a triangle with the heavy atom in the center; if it has a sp hybrid orbital, the heavy atom and the two bonded atoms tend to form a triangle with the three atoms on its vertices.

In our method, H-atoms are initially placed based on the local geometry, which is determined by the hybrid orbital of the heavy atom to which the hydrogen atom is connected. In general, three constraints are required to fix the spatial position of an H-atom. Two of them are the bond length and the bond angle, which are constant and taken from the CHARMM22 force field [28]. The third constraint is determined based on the classes of the given H-atom; H-atoms are classified based on the type of the hybrid orbital and the number of H-atoms connected to the central heavy atom (see Table 1).

**Table 1:** Classification of hydrogen atoms, and their bond lengths and locations.

Class	Schematic figure	Bond length (Å)^a^	Location
sp3H3	-CH3, -NH3	1.111/1.040	Ala, Ile, Leu, Met, Thr, Val, Lys, N-term (not Pro)
sp3H2	>CH2, -NH2	1.080/0.997	All except Ala, Thr, Val, and –NH2 only for Pro in N-term
sp2H2	-NH2	1.000	Arg, Asn, Gln
sp3H1	>CH-	1.083	All except Gly
sp2H1	≥CH, >NH	1.070/0.976	Arg, His, Phe, Trp, Tyr and all peptide plane (not Pro)
spH1	-OH	0.960	Ser, Thr, Tyr

a When two values are shown, the first is the bond length of C-H; the second is that of N-H.

**Figure 1:**
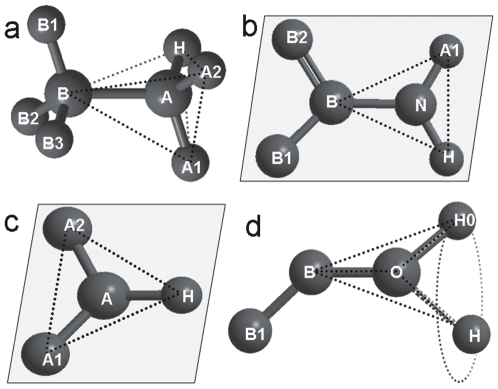
Illustration of hydrogen atom placement based on local geometry. (a) The hydrogen atoms are bonded to the heavy atom A with an sp3 hybrid orbital; (b) and (c) the local geometry for sp2 hydrogen atoms; (d) local geometry for sp hydrogen atoms. The labels A and B denote the position which may hold C, N or other atoms in the protein chain; the labels A1, A2 and B1, B2, B3 represent atoms or atomic groups. The excluded volumes are ordered as A1≥A2≥H, and B1≥B2≥B3. The dotted lines indicate the geometry determined by the hybrid orbital. In (d), H0 is at the initial position with a trans-conformation; H is at the position obtained after considering non-bonded interactions.

In Figure 1, we present an illustration of how the local geometry is determined by the hybrid orbital. We label the central heavy atom under consideration as A and the neighboring central heavy atom as B, with A1, A2, B1, B2 and B3 denoting the groups connected to these central atoms, where for the exclusive cases the atoms are labeled with their element symbol. The atoms involved in an sp3 hybrid orbital have a preference for a staggered conformations because this state ensures the minimum local steric repulsion between the atoms [29], [30]. Therefore, we place H-atoms in the sp3H3 class in a staggered conformation (labeled A1, A2 & H in Fig 1a) without further optimization, although they may have rotational freedom around the A–B bond. To assign the position of sp3H2 H-atoms, we first identify the tetrahedron centered at A with two of its vertices at B and the heavy atom A1, and then put the two H-atoms at the remaining vertices of the tetrahedron (A2 and H in Figure 1a), while retaining the standard bond lengths and bond angles. In the case of sp3H1 H-atoms, because the three heavy atoms at B, A1 and A2 form three vertices of the tetrahedron centered at A, the sp3H1 H-atom is placed at the remaining vertex of the tetrahedron (H in Figure 1a), with the standard parameters.

For constructing the sp2H2 and sp2H1 H-atoms, we first decide on the orientation of the conjugated plane or the aromatic ring with respect to the neighboring heavy atoms; the normal vector of the conjugated plane is determined by taking the cross product of two vectors between the heavy atoms. For the sp2H2 H-atoms (illustrated in Fig. 1b), the normal vector of the conjugated plane is the cross product of the unit vectors B→N and B2→B; then the two H-atoms are placed at positions A1 and H, which are within the conjugated plane respected to the B→N vector with the exact bond angle from CHARMM22 force field. For sp2H1 H-atoms, two conformations are possible. The first is to place the H-atom in the peptide plane as illustrated in Fig. 1b, where A1 and B1 represent the alpha carbon atoms. The position of H in this case is decided by using the same method as the one used to determine the position of the sp2H2 H-atoms while holding the trans-conformation. The second possible conformation is for a hydrogen in an aromatic ring, as illustrated in Fig. 1c. The normal vector of the conjugated plane is defined by the cross product of the unit vectors of A1→A and A2→A; and the H-atom is then placed in the conjugated plane along the vector satisfying the bond length and the bond angle.

H-atoms in the spH1 category constitute less than 2% of all H-atoms in proteins. However, the placement of spH1 H-atoms is usually less accurate than that of other H-atoms due to the fact that these H-atoms have a rotational freedom and can be located at any position around the circle in a cone (see Figure 1d). To decide on the position of spH1 atoms, we initially place the H-atoms in a trans-conformation using a similar protocol to the spH1 atoms (H0 in Fig. 1d), and then relocate them based on the global minimum of the energy function
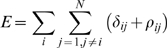
(1)where *i* runs through all spH1 H-atoms and *N* is the total number of atoms in the protein chain. *δ*_ij_ = 10(*δ*_i_+*δ*_j_−*r*_ij_) when *r*_ij_<*δ*_i_+*δ*_j_; otherwise equals to zero. *ρ*_ij_ = *ρ*_i_ *ρ*_j_ when *r*_ij_≤4 Å; otherwise equals to zero. Here *δ*_i_ and *ρ*_i_ are the van der Waals radius and the partial charge of the *i*th atom from the CHARMM22 force field [28], and *r*_ij_ is the distance between the *i*th and the *j*th atom. The first term in Eq. (1) is used to minimize steric clashes of the *i*th H-atom with other atoms, while the second term accounts for the electrostatic interactions and guides atoms of opposite partial charges to be placed close to each other. Since the hydrogen bond donor and acceptor atoms have opposite partial charges, minimization of *ρ*_ij_ tends to encourage the formation of more hydrogen bonds. We search the conformational space by rotating the dihedral angle B1-B-O-H in a 10° interval starting from the initial position and finally adopt the position on the cone with the smallest energy.

HAAD is a standalone program written in FORTRAN90. The average CPU time required for constructing all H-atoms in a protein structure with ∼200 amino acids is 0.06 seconds on a 2.6 GHz AMD processor machine, which is about 8 times faster than REDUCE (0.46 seconds) and 18 times faster than HBUILD (1.09 seconds) according to our test on 230 protein structures. The on-line server, the executable and source code of the HAAD program are freely available at http://zhang.bioinformatics.ku.edu/HAAD/.

## Materials

For a given protein structure with fixed heavy atom positions, the possible variation in H-atom positions is relatively small, especially compared to the possible topology changes resulting from changing the backbone conformation. Therefore, high-resolution structures including H-atoms are essential for evaluating hydrogen addition algorithms. For this purpose, two sets of experimental protein structures containing chains of at least 30 residues with explicitly solved H-atoms were selected from the PDB. The first set includes ultra-high-resolution protein structures solved by X-ray crystallography experiments with a resolution better than 1.0 Å (Table 2); the second set includes structures solved by high-resolution neutron diffraction, in which the relative orientation of the groups containing H-atoms are accurately determined [31].

**Table 2:** List of the proteins solved by high-resolution X-ray and neutron diffraction experiments used for analysis.

PDB	Length	Resolution (Å)	No. of hydrogen atoms
*X-ray*			
1ab1	46	0.89	302
1dy5	123	0.87	889
1fy5	217	0.81	1413
1g66	207	0.90	1343
1gci	269	0.78	1731
1i1w	302	0.89	2114
1m40	263	0.85	1716
1muw	386	0.86	2900
1vyr	363	0.90	2442
1p9g	40	0.84	242
1pq5	224	0.85	1497
1ssx	170	0.83	1173
1ucs	64	0.62	518
1x6z	119	0.78	859
1xvo	224	0.84	1504
1yk4	52	0.69	367
2b97	140	0.75	985
2h5c	170	0.82	1161
2h5d	173	0.90	1169
2p74	522	0.88	3804
2pve	156	0.79	1101
3pyp	125	0.85	928
*Neutron diffraction*			
1wq2	131	2.4	786
1l2k	151	1.5	967
1xqn	237	2.5	1749
1lzn	129	1.7	695
1ntp	223	1.8	1433
1iu6	51	1.6	335
2efa	30	2.7	205
2gve	388	2.2	2720
1vcx	53	1.5	348
1io5	129	2.0	696
2mb5	153	1.8	974
5rsa	124	2.0	693
1c57	237	2.4	1749
1cq2	153	2.0	1230
1gkt	334	2.1	2015

To assess the accuracy of predicted H-atom positions on these two sets of proteins, all the H-atoms in these protein structures were first removed, and then added using HBUILD (from CHARMM) [20], [21], REDUCE [19] and HAAD. We choose HBUILD and REDUCE for comparison because they are widely used and are among the most accurate methods based on recent assessments [18]. Because REDUCE may flip the side chains of Gln, Asn, and His to resolve clashes during H-atom construction which results in additional errors when assessing the models by REDUCE, to have a fair comparison, we excluded those proteins from our benchmark set, in which side chains were flipped, by checking whether the root mean square deviation (RMSD) of all heavy atoms is equal to zero between the structures before and after adding the hydrogen by REDUCE. Finally, 22 X-ray structures and 15 neutron diffraction structures were selected for the comparison and analysis. It is worth mentioning that in the analysis of the protein structures solved by the neutron diffraction, we exclude deuterium atoms in the experimentally solved structures from the comparison with the predicted H-atom positions, because deuterium atoms have different bond lengths and van der Waals radii than H-atoms.

Protein structures solved by NMR are usually determined by satisfying the spatial distance restraints [32] which can be derived from the proton-proton distances in the Nuclear Overhauser Effect (NOE) data. Because of the limited number of NOEs, there are usually a number of NMR models in the PDB files which fit equally well to the NOE data and thus result in uncertainty in the heavy atom coordinates. Especially, the H-atoms in NMR are usually determined by running existing H-adding software and the accuracy of the software programs can be questionable. Thus, we do not consider the NMR models as *objective* criterions for examining the developed H-adding algorithms. Instead, we test the algorithms based on the original NOE data with proton-proton distances and the corresponding X-ray diffraction structures. For this purpose, we collected 13 proteins, as shown in Table 3, from the PDB which have been solved by both NMR (for collecting NOE) and X-ray crystallography, and have their NOE data deposited in BMRB [33]. We first rebuild all the H-atoms based on the X-ray heavy atom structures, and generate an inter-proton distance map which is then compared with the original NOE distance restraints. Although the X-ray structures and the NOE data are obtained in different environments and may reflect structural diversity and have different resolutions, the assumption here is that the correctly positioned H-atoms should, on average, have the maximum convergence with NOE proton distance map, because they are from the same proteins. It needs to be mentioned that in the comparison of the distance map with the NOE data, we only consider those NOE distance restraints which have a mean distance of no more than 5 Å, because the Nuclear Overhauser effect above this distance becomes relatively weak [34].

**Table 3:** List of proteins having both an X-ray structure and NOE data deposited in PDB, which are used for analysis.

PDB ID in NMR	PDB ID in X-ray	Length	RMSD (Å)^a^	Resolution (Å)^b^	*N*_NOE_ c
1vre	1jf4	147	1.333	1.40	2097
1jor	1ey4	134	2.792	1.60	1596
1bla	1bfg	126	0.976	1.60	2196
1kdf	1msi	64	0.826	1.25	1197
1ikm	3il8	68	4.733	2.00	892
3gbl	1pgb	56	0.541	1.92	671
3ci2	2ci2	63	1.262	2.00	944
1eq0	1hka	158	3.182	1.50	2856
3phy	1gsv	121	1.932	1.75	1145
1r63	1r69	63	0.764	2.00	531
1jnj	1lds	96	3.450	1.80	696
3mef	1mjc	68	1.529	2.00	421
1jv9	6pti	55	0.690	1.70	534

a RMSD of all the heavy atoms after superposing the NMR and the X-ray structures.

b Resolution of the X-ray structures.

c Number of NOE distance restraints with the mean proton-proton distance below 5 Å.

To evaluate the accuracy of hydrogen positions using the NOE distance restraints, the ratio of matched proton-proton pair (*f*_match_) is counted through the definition of
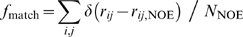
(2)where *r_ij_* is the distance between the *i*th and the *j*th H-atoms predicted by the hydrogen addition programs based on the heavy atom of X-ray structures; *r_ij_*_,NOE_ ( = 5 Å) is a mean distance cutoff of the NOE restraint data for the corresponding atom pairs and *N*_NOE_ is the number of NOE distance restraints with the mean proton-proton distances below 5 Å as shown in Table 3. The step function *δ*(*x*) = 1 if *x*≤0; otherwise it is equal to 0.

## Results

### Deviation of predicted H-atom from X-ray and neutron diffraction structures

RMSD is commonly used as a direct measure for assessing the accuracy of the predicted H-atoms in comparison with those in high-resolution experimental structures. Instead of an all-atom superposition as done in usual RMSD calculation [35], we first superimpose the structure of the heavy atoms and then directly calculate the root mean square of the distances between corresponding H-atom pairs.

Table 4 summarizes the RMSD of the H-atoms added by the three different methods used in our study. It shows that the H-atoms added by HAAD have a lower RMSD to the experimental structures than those added by HBUILD and REDUCE in all the H-atom categories except spH1. For the spH1 atoms, the average RMSD from HAAD (1.111 Å) is lower than that from HBUILD (1.217 Å) but slightly higher than that from REDUCE (1.094 Å). The average RMSD for all 46,753 H-atoms is 0.208 Å, 0.234 Å, and 0.282 Å for HAAD, REDUCE, and HBUILD, respectively.

**Table 4:** Summary of the accuracy of hydrogen atoms placement by different methods as compared to high resolution X-ray and neutron diffraction structures.

Hydrogen	No. of H-atoms	RMSD (Å)
		HBUILD	REDUCE	HBUILD
Polar	7,570	0.424	0.388	0.379
Non-polar	39,183	0.246	0.190	0.154
sp3H3	10,733	0.292	0.292	0.249
sp3H2	17,202	0.275	0.142	0.101
sp2H2	1,657	0.245	0.222	0.177
sp3H1	7,908	0.113	0.116	0.097
sp2H1	8,479	0.139	0.142	0.107
spH1	774	1.217	1.094	1.111
All/Average	46,753	0.282	0.234	0.208

**Figure 2:**
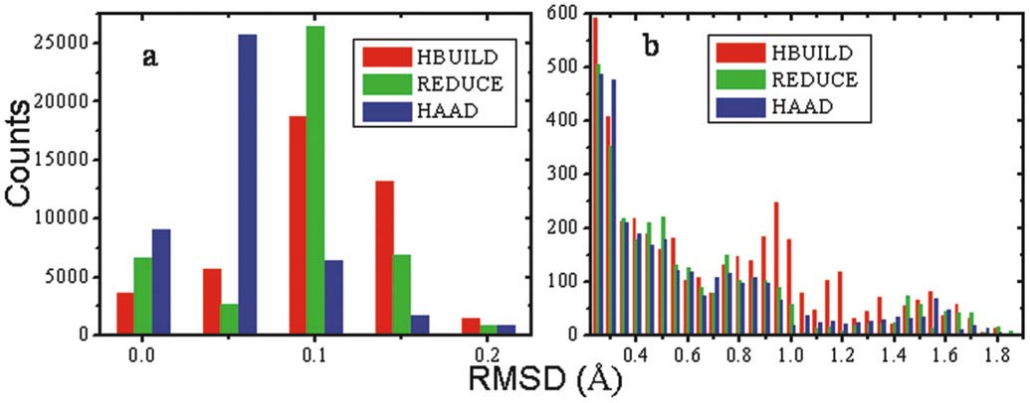
The RMSD distribution in the small deviation (a) and the large deviation category (b).

In Figure 2, we split the H-atoms added by each algorithm to all structures in our test sets into two categories: those having a small deviation (distance≤0.2 Å) and those having a large deviation (distance>0.2 Å) from their respective native positions. In the small deviation category (Figure 2a), all the three programs have an appreciable accuracy, with 93.5% of H-atoms added by HAAD falling in this category, while 92.3% and 91.2% H-atoms by REDUCE and HBUILD are in this category, respectively. At a more restrictive distance cutoff of RMSD≤0.1 Å, the performance difference becomes more pronounced, with 88.0% of H-atoms added by HAAD falling in this category, while only 76.6% and 59.9% of H-atoms predicted by REDUCE and HBUILD are in this category, respectively.

In the large deviation category (Figure 2b), the three methods show similar distributions. The largest observed deviations reaches 1.85 Å got 9 H-atoms placed by REDUCE, 8 of them belong to the spH1 class. The profile of the RMSD distribution for all the H-atoms is in agreement with the distribution of the mean square displacements of H-atoms in experimental structures [13], [18]. Overall, HAAD has the ability to place H-atoms with a smaller deviation from their positions in the experimentally solved high-resolution structures than other programs.

**Figure 3:**
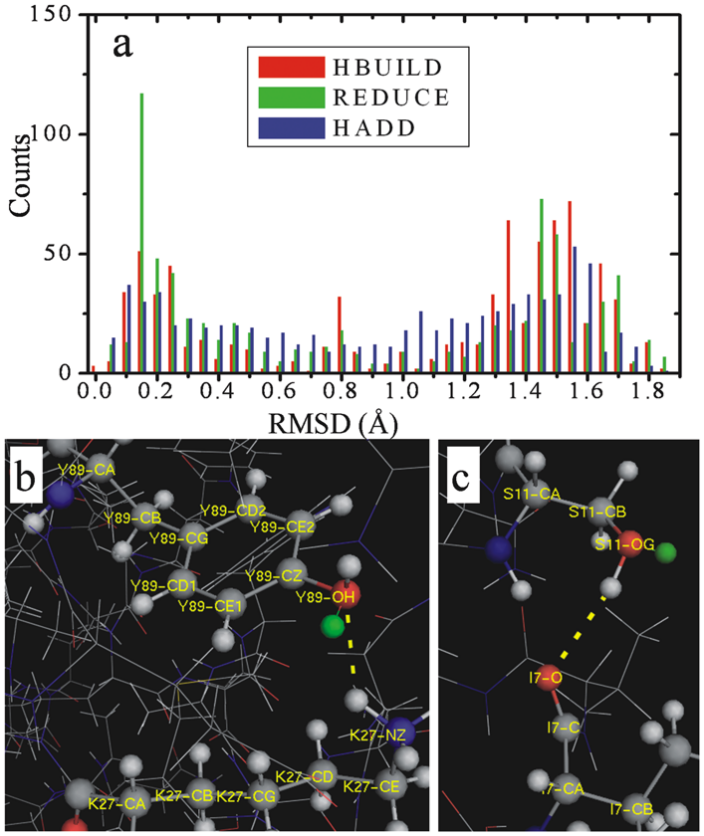
The RMSD distribution of spH1 hydrogen atoms and examples. (a) The RMSD distribution in the spH1 category. (b) An example from 1gci, showing the OH group in Y89 as an acceptor of a hydrogen bond with the NZ atom in K27. (c) An example from 1ab1, showing the OH group in S11 as a donor of a hydrogen bond with the O atom in I7. The yellow dashed line indicates the hydrogen bond; the grey, red, blue and white balls represent C, O, N and H atoms, respectively. The green sphere indicates the favorable position of the hydrogen as corresponding to the local geometry, which becomes unfavorable because of the formation of hydrogen bonds.

To find out which atoms contribute most of the large deviations, we show the distance distribution of the spH1 H-atoms in Figure 3a. For all the H-atoms in the 37 structures with a distance ≥1.0 Å, 422 out of 454 H-atoms rebuilt by HAAD, similarly 481 out of 1031 by HBUILD, and 368 out of 475 by REDUCE, belong to the spH1 class. These data again show that the spH1 H-atoms are the major contributions to the large deviation category, and that spH1 is the most difficult class of H-atoms to be accurately predicted.

In addition to the fact that the spH1 H-atoms have a large degree of positional uncertainty according to the hybridization model shown in Figure 1d, we assume that the relocation of H-atoms in –OH groups due to the formation of hydrogen bonds is another reason contributing to lower accuracy of predicted spH1 H-atom positions, The –OH group can serve either as a donor or an acceptor or both in a protein chain and the hydrogen bonding energy is favorable enough to change the stereochemistry and conformation of this group. In fact, we observed a number of cases where H-atoms are obviously relocated due to the formation of hydrogen bonds. Figure 3b shows one example, the hydrogen in the -OH group of Y89 (TYR) of the protein 1gci, which has been driven away from the aromatic conjugated ring plane (i.e. the favorable position corresponding to the local geometry as shown by the green sphere) to decrease the steric repulsion from the donor NZ in K27 (LYS), with which a hydrogen bond is formed (indicated by the yellow dashed line). Figure 3c is another example, from protein 1ab1, where the hydrogen in OG group of the S11 (SER) side chain is drawn away from the position corresponding to the minimum of the local steric repulsion (green sphere), because the OG atom serves as a donor of a hydrogen bond whose acceptor is the O atom in I7 (ILE). Since hydrogen bonds involving –OH groups can be formed both in the buried core region (with other polar groups) and ath the exposed protein surface (with solvent molecules), the position of an spH1 H-atoms does not depend on whether the –OH group is buried or not.

### Atomic clashes of predicted H-atoms with other atoms

The number of atomic clashes between the added H-atoms and other heavy atoms is an another important evaluation criterion to assess the quality of hydrogen addition algorithms [36]. Two atoms clash when the distance between them is less than the sum of their van der Waals radii. Ideally, the atoms in the native structures have no (or very few) clashes, suggesting that structures with fewer atom clashes should be more reliable and native-like.

The normalized number of clashes made by H-atoms in category T in a protein can be calculated by
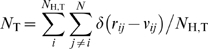
(3)where *v*_ij_ equals to the sum of the van der Waals radius of the *i*th and the *j*th atoms with values taken from the CHARMM22 force field (see ‘par_all22_prot.inp’ in the CHARMM22 package). *δ*(*x*) = 1 if *x*<0, otherwise equals 0. *N*_H,T_ is the number of H-atoms in category T, where T may denote all H-atoms (“all”), polar H-atoms (“polar”) and non-polar H-atoms (“non-polar”). When counting the number of clashed atom pairs, atom pairs with strong chemical geometry restraints, i.e. fewer than three covalent bonds apart, are excluded. Because the polar H-atoms can easily undergo an exchange with the solvent [13] and the properties of hydrogen and deuterium are different, we also exclude those atom pairs from the comparison that involve deuterium atoms in the neutron diffraction structures.

**Figure 4:**
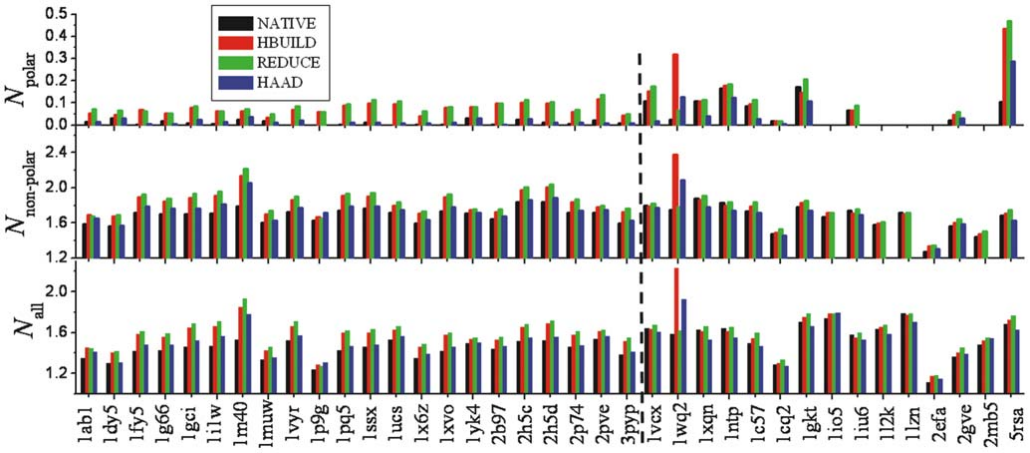
The average number of atom clashes made by hydrogen atoms in various categories, in models of 37 protein structures. The dashed line marks the boundary between X-ray (left) and neutron diffraction structures (right).

The number of atomic clashes between the predicted H-atoms and other atoms for all the 37 high resolution structures are shown in Fig. 4 and the average values are summarized in Table 5. Some of the structures solved by neutron diffraction have an *N*_polar_ equal to 0 because no polar H-atom is compared in these structures. On average, for all the H-atoms, the experimental structures have the lowest average number of atomic clashes, i.e. *N*_all_ = 1.48. The number of clashing atoms in structures generated by HAAD is 2% higher than that in the experimental structures, but 5% lower than that in models from HBUILD and 6% lower than that in models from REDUCE.

**Table 5:** Comparison of the average number of atom clashes and its standard deviation (in parentheses) of the predicted hydrogen atoms in the models built by different methods.

Hydrogen	Experimental structures	HBUILD	REDUCE	HAAD
Polar	0.03 (0.04)	0.08 (0.08)	0.09 (0.07)	0.04 (0.05)
Non-polar	1.75 (0.13)	1.86 (0.20)	1.88 (0.18)	1.80 (0.16)
All	1.48 (0.14)	1.59 (0.18)	1.60 (0.15)	1.51 (0.15)

### Consistency of H-atom predictions with NOE distance restraints

**Figure 5:**
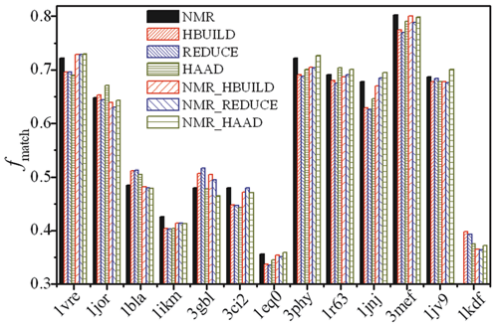
The number of hydrogen atom pairs matching the NOE proton-proton distance restraints in models of 13 proteins. Models are from in the NMR structures, and the structures built by the three methods based on either X-ray or NMR heavy-atom structures. For 1 kdf, the hydrogen in the NMR model is not available.

In Figure 5, we present the comparison of predicted H-atoms with data from NMR experiments. Because H-atoms in NMR models are usually added based on existing H-adding algorithms, to eliminate the algorithm-dependent bias, we compare our H-adding prediction directly with the original NOE proton-proton distance data, where the structure models with the H-atoms are reconstructed by HAAD, HBUILD and REDUCE based on the X-ray heavy-atom structure of the same proteins. As shown in Eq. (2), *f*_match_ is defined as the number of matches between NOE restraints and the predicted H-atom distances divided by the number of NOEs. *f*_match_ as calculated based on the NMR structural models is also shown for a reference comparison. For proteins with multiple NMR models, the model which has the minimum RMSD to the X-ray structure is presented.

Despite considerable systematic errors due to the fact that the proteins are solved in different environments by NMR and X-ray crystallography, there are obvious differences between the models made by the three different methods. From the 13 proteins that were used in this analysis, HBUILD has 4 (1 ties with HAAD), REDUCE has 4 (1 ties with HBUILD), and HAAD has 7 (1 ties with HBUILD) cases with the highest *f*_match_ values. The average *f*_match_ for all the 12 proteins (except for 1 kdf that has no H-atoms in the NMR structure) are 0.598, 0.584, 0.583 and 0.588 for the NMR model, and the structure models by HBUILD, REDUCE and HAAD, respectively. The *f*_match_ for 1 kdf is comparable and is equal to 0.398, 0.393 and 0.376 for HBUILD, REDUCE and HAAD, respectively.

The main reason of choosing X-ray diffraction structures instead of NMR models as starting model for constructing H-atoms in the above experiment is that the X-ray structure is much less program-dependent while NMR models are usually built based on molecular simulations under NOE restraints. In case that the number of NOEs is limited, several models can be generated. In the right columns of Figure 5, we also compare the NOE data with the H-atoms predicted on the NMR heavy atom structures that are closest to the X-ray structure. Similarly, HBUILD gets 3 (1 ties with REDUCE), REDUCE gets 2 (1 ties with HBUILD) and HAAD gets 8 cases which have the highest *f*_match_ values. The average *f*_match_ in all the 12 proteins (except for 1 kdf) are 0.595, 0.594 and 0.598 for the H-atom models built by HBUILD, REDUCE and HAAD. These data show that the H-atoms in the models build by HAAD have a greater consistency with the NOE distance restraint data that the other two programs.

## Discussion

In general, non-polar H-atoms have a smaller RMSD than polar H-atoms in all three methods. This can be explained by the large positional uncertainty of polar H-atoms induced because of their hydrogen-bonding capability. Quantitatively, the free energy cost of moving a H-atom from the staggered conformation to an eclipsed conformation is around 3.0 kcal/mol [29]. But the free energy gained by forming a hydrogen bond in the polar H-atoms is about 5.0 kcal/mol [37]. This renders the polar H-atoms to readily depart from their standard staggered conformations when a hydrogen bond can be formed, making the emplacement of H-atoms based on local geometry a formidable task. The accurate prediction of polar H-atom positions requires further consideration of both local steric repulsion and non-local hydrogen-bonding networks.

On the other hand, for non-polar H-atoms, the average free energy gain for a hydrophobic interaction pair is about 0.18 kcal/mol [37], which is too weak to move the H-atoms away from their most stable rotational conformation (with minimum local steric repulsion), suggesting that non-polar H-atoms are most likely located close to the position determined by the hybridization state of the central heavy atom. This corresponds to the way of placing non-polar H-atoms in our method, and is also supported by protein structures obtained from neutron diffraction [38].

Among the different categories, the spH1 H-atoms have the largest deviation from the native position, and all three methods failed to achieve an average RMSD below 1.0 Å. This is not surprising considering the fact that the spH1 H-atom positions have the largest degree of uncertainty according to the hybridization model (see Figure 1d). The positions of the sp3H3 H-atoms are the second hardest to predict because they have a rotational freedom around the sigma bond (B-A in Figure 1a). In most of the experimental structures, they occupy positions that are close to be not exactly at the positions corresponding to a staggered conformation. For the other four hybridization categories, almost all the H-atoms can be correctly placed by HAAD within an average deviation below 0.2 Å.

With regard to the atomic clashes of H-atoms with others, it is observed that the absolute number of atomic clashes involving non-polar H-atoms is much higher than the number of clashes made by polar H-atoms; this is because non-polar H-atoms are mostly located in the hydrophobic core, which is usually tightly packed [39], and thus have a higher chance to clash with other atoms. On the other hand, the polar H-atoms are mostly in the interface or on the surface, where the atomic packing density is lower than in the core region. Moreover, the polar H-atoms frequently mix with charged groups where the electrostatic repulsion acts against atomic packing. Therefore, the non-polar H-atoms have a smaller free space to accommodate to than polar H-atoms, which result in more atom clashes in the non-polar H-atoms than that in the polar ones.

It has been reported that the length of bonds between hydrogen and heavy atoms are systematically underestimated in X-ray diffraction [39], [40]; this may be partially the reason why there are still some atomic clashes in the experimental structures. Overall, the number of clashes in the HAAD models is closer to that observed in the experimental structures than the numbers from REDUCE and HBUILD models, which demonstrates that the method we used for constructing H-atoms is more efficient in reducing the atom clashes.

### Summary

We developed a new algorithm, HAAD, for quickly predicting the positions of H-atoms in protein structures. The method is built on the basic theory of orbital hybridization, followed by the optimization of steric repulsion and electrostatic interactions.

HAAD constructs H-atoms in protein structures with an appreciable accuracy. In three independent tests based on experimental data from ultra-high-resolution X-ray structures, neutron diffraction experiments, and NOE proton-proton distance restraint data, the overall accuracy of the hydrogen positioning by HAAD is consistently higher than that of other methods used for hydrogen construction. The average RMSD of H-atoms placed by HAAD from their corresponding positions in the ultra-high-resolution experimental structures is ∼26% lower than that obtained with HBUILD, a subroutine for hydrogen construction in CHARMM [21], and 10.7% lower than that by REDUCE [19]. When comparing the NOE restraint data with the hydrogen positions built from both the X-ray structures and the NMR models of the same proteins, the models built by HAAD have a higher number of H-atom pairs consistent with the original NOE data than models built by other methods. Although we are aware of the fact that positions of H-atoms in most experimental structures have a high uncertainty compared with the accuracy we addressed here, we believe that our evaluations using a large-scale data (46,753 H-atoms and 15,776 NOE proton-proton distances), including ultra-high-resolution structures, should provide a statistically meaningful differentiation between the respective performances of the tested methods.

As an additional assessment, the number of steric clashes in the HAAD models is relatively lower than in other models. Because the non-polar H-atoms are usually located in the densely packed hydrophobic core, they have a much higher number of clashes than the polar H-atoms which tend to be located on the surface. The number of total clashes in the HAAD models is only 2% higher than the experimental structures, and 5–6% lower than that in models by HBUILD and REDUCE.

In general, the accuracy of predicted polar H-atoms is lower than that of non-polar H-atoms; the accuracy for hydrogen in –OH groups is the lowest among all the different categories of H-atoms. This is mainly due to the fact that the hydrogen-bonding interactions of the polar and spH1 H-atoms with other charged groups (including solvent molecules) tend to drive the H-atoms away from the locally optimal position with minimum steric repulsion. Therefore, further refinement of the global hydrogen-bonding networks, as well as including the interactions with water molecules, may help improve the accuracy of adding polar and spH1 H-atoms, although it will require more CPU cost; a new version of HAAD along this line is in development. Nevertheless, the encouraging results in improving the hydrogen accuracy and the ability of quickly constructing H-atoms should make the current version of HAAD an important tool for detailed studies of protein structure and function, especially in large-scale and atomic-level simulations where the positions of hydrogen atoms need to be quickly and accurately determined.
